# Use of Resazurin To Rapidly Enumerate *Bdellovibrio* and Like Organisms and Evaluate Their Activities

**DOI:** 10.1128/spectrum.00825-22

**Published:** 2022-06-13

**Authors:** Hyochan Jang, Wonsik Mun, Seong Yeol Choi, Robert J. Mitchell

**Affiliations:** a School of Life Sciences, Ulsan National Institute of Science and Technology (UNIST), Ulsan, South Korea; University of Guelph

**Keywords:** bdellovibrios, predatory bacteria, resazurin, fluorescence assays

## Abstract

A method to rapidly quantify predatory bacterial cell populations using resazurin reduction to resorufin and its resulting fluorescence kinetics (dF/dt) are described. The reliability of this method to measure the predatory populations was demonstrated with the type strain, Bdellovibrio bacteriovorus HD100, as well as B. bacteriovorus 109J and two natural isolates, *Halobacteriovorax* strains JA-1 and JA-3, with clear correlation when densities were between 10^7^ and 10^9^ PFU/ml. Resazurin was also used to evaluate how B. bacteriovorus HD100 and *Halobacteriovorax* strain JA-1 respond to harmful conditions, i.e., exposure to sodium dodecyl sulfate (SDS), with both the dF/dt and PFU/ml indicating *Halobacteriovorax* strain JA-1 is more sensitive to this surfactant. Tests were also performed using media of different osmolalities, with the dF/dt values matching the 24-h predatory activities reasonably well. Finally, this method was successfully applied in near real-time analyses of predator-prey dynamics and, when coupled with SDS, was capable of differentiating between the predatory and prey populations. All of these tests serve to prove this method is (i) very rapid, needing only 15 min from start to finish; (ii) very reliable with different predatory bacterial species; and (iii) very versatile as it can be easily adapted to measure predatory numbers and activities in a range of experiments.

**IMPORTANCE** *Bdellovibrio* and like organisms are predatory bacteria that are capable of attacking, killing, and consuming many bacterial pathogens, including multidrug-resistant strains. These qualities have led to them being labeled as “living antibiotics.” Research work with these remarkable strains, however, has been hampered by long growth times needed to quantify the predatory populations through plaque assays, which typically take 4 days to develop. Here, we describe a fluorescence-based method using the conversion of resazurin (low fluorescence) to resorufin (high fluorescence) after it is reduced by the predators’ NADH. Not only do we show that the fluorescence correlates strongly with the predatory concentration and that we can use it to evaluate if the predators are viable, but the entire procedure from start to finish takes only 15 min, drastically reducing the time researchers need to quantify the predatory numbers. Employing this technique will greatly advance research related to predatory bacteria and their potential applications.

## OBSERVATION

Intraperiplasmic predatory bacterial strains, of which Bdellovibrio bacteriovorus is the best known, are a group of Gram-negative bacteria that actively attack and consume other Gram-negative bacterial species from within (1, 2). Since their discovery 6 decades ago by Stolp and Starr (3), work has continuously progressed to characterize their activities, with current emphasis focusing on their ability to predate on bacterial pathogens (4, 5), including antibiotic-resistant strains (6, 7). In all of these studies, however, top agar plates were used to enumerate the predatory populations, a protocol that requires typically 3 to 4 days.

This is an undeniable bottleneck as it delays interpreting experimental results. To address this, researchers have adopted a variety of protocols and genetic tools, including the use of fluorescent predatory strains (8, 9), bioluminescent prey (10), SYBR green (11), or flow cytometry (12). While each offers some benefits, they also have their own limitations, including the need for genetic engineering of the predator or prey, which may not always be possible, or problems differentiating between live and dead predators.

**FIG 1:**
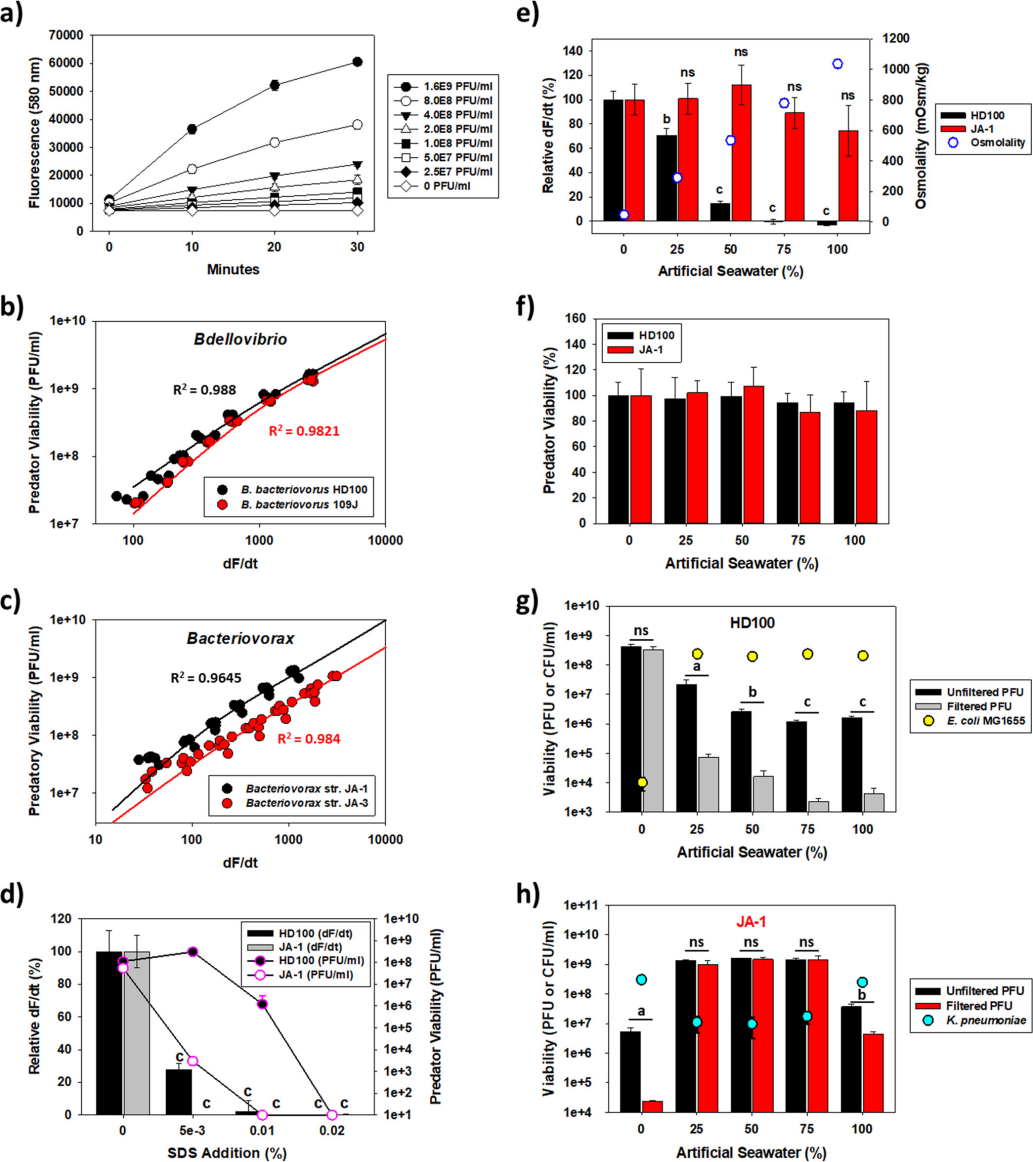
Rapid enumeration of predatory bacteria using resazurin. (a) Kinetic plot showing the increase in fluorescence based on the B. bacteriovorus HD100 concentration. The data show the largest increase for all samples was during the first 10 min, and consequently, the dF/dt was always calculated using the difference between the 0-min and 10-min readings (*n *= 3). (b) Plot of the dF/dt values for the *Bdellovibrio* predatory strains against their cell densities, showing the high degree of correlation when the predatory numbers were between 10^7^ and 10^9^ PFU/mL (*n *= 3). (c) Plot of the dF/dt values for the *Halobacteriovorax* predatory strains against their cell densities, once more showing the high degree of correlation when the predatory numbers were between 10^7^ and 10^9^ PFU/mL (*n *= 6). (d) The resorufin fluorescence drops when the predatory cultures are killed by SDS. The bacterial predators were incubated with the different SDS concentrations for 1 h before adding resazurin. The results show the dF/dt values can be used to evaluate when the predators are killed under certain conditions. c, *P* < 0.0001 (*n *= 3). (e) Impacts of medium osmolality on the dF/dt values from B. bacteriovorus HD100 and *Halobacteriovorax* JA-1, indicating the former is negatively affected while the latter is resilient. The bacterial predators were incubated with the different media for 1 h before adding resazurin. ns, not significantly different; b, *P* < 0.001; c, *P* < 0.0001 (*n *= 3). (f) One-hour viabilities for each predator. After incubation of the predators at the different osmolalities in the experiment in panel e, their viabilities were determined using top agar plates, showing neither predator had its viability negatively impacted compared against 0% ASW (*n *= 3). (g) Overnight predation results for B. bacteriovorus HD100, showing the predatory activities against E. coli in each osmolality correlated well with the dF/dt values found in panel e. ns, not significantly different; a, *P* < 0.05; b, *P* < 0.001; c, *P* < 0.0001 (*n *= 3). (h) Overnight predation results for *Halobacteriovorax* JA-1, showing the predatory activities against Klebsiella pneumoniae in each osmolality correlated well with the dF/dt values found in panel e, except for the 0% ASW, where predation did not occur. ns, not significantly different; a, *P* < 0.05; b, *P* < 0.001 (*n *= 3).

In response, we describe here the use of resazurin to rapidly quantify predatory populations. Resazurin is a membrane-permeative compound that reacts with the intracellular NADH pool and is reduced to resorufin, a stably fluorescent compound (13). While resazurin has been used extensively to evaluate bacterial viabilities, particularly in antibiotic susceptibility assays (14–16), it has never been employed with *Bdellovibrio* and like organisms (BALOs). As shown in Fig. 1a, in tests with the type strain B. bacteriovorus HD100, resazurin was quickly reduced to resorufin by this bacterium in a concentration-dependent manner. As the greatest fluorescence kinetic change occurred in the first 10 min (i.e., dF/dt), this was used to evaluate the potential use of resazurin to enumerate this predator. When the measured dF/dt values were plotted against the B. bacteriovorus HD100 viabilities, we found a clear dose-dependent relationship (Spearman’s *R*^2^ = 0.988; standard deviation, 22.5%) when the predatory densities were between 10^7^ and 10^9^ PFU/mL (Fig. 1b). This was true not only for B. bacteriovorus HD100, though, as similar results were also obtained with three other predatory strains, i.e., B. bacteriovorus 109J (*R*^2^ = 0.9821; Fig. 1b) and two newly isolated *Halobacteriovorax* strains (JA-1 and JA-3 [Fig. 1c and see Fig. S1 in the supplemental material] [*R*^2^ = 0.9645 and *R*^2^ = 0.984, respectively]).

These results prove resazurin can be used to quickly and reliably estimate predator populations within only 15 min. We next raised the question if resazurin can also be used to gauge how predators respond under other conditions. For instance, B. bacteriovorus is known to be highly sensitive to surfactants, particularly sodium dodecyl sulfate (SDS) (17), but the sensitivity of *Halobacteriovorax* sp. was not evaluated. Consequently, one of each species (i.e., B. bacteriovorus HD100 and *Halobacteriovorax* strain JA-1) was exposed to different concentrations of this surfactant for 1 h prior to adding resazurin. As shown in Fig. 1d, the resorufin fluorescence dropped precipitously when SDS was present for both strains, but more quickly for *Halobacteriovorax* strain JA-1. This sensitivity profile matched the viability results very nicely, with B. bacteriovorus HD100 being more resistant to SDS until 0.02%, at which neither predator survived.

Encouraged by these results, we expanded our focus to osmolality. As reported previously, B. bacteriovorus HD100 is also sensitive to the medium osmolality, with predation completely blocked when the osmolality is 300 mosmol/kg or higher (18). As *Halobacteriovorax* strain JA-1 was isolated using a 30% artificial seawater (ASW) medium, where the osmolality was around 350 mosmol/kg, we were curious if these differences can be captured using resazurin. Consequently, both strains were exposed to different medium osmolalities (from 40 to 1,040 mosmol/kg) for 1 h. Agreeing with the study by Im et al. (18), the dF/dt values for B. bacteriovorus HD100 dropped as the osmolality increased and eventually reached a low plateau at 75% ASW, or 780 mosmol/kg (Fig. 1e). For *Halobacteriovorax* strain JA-1, however, the medium osmolality had no significant impact, although the resorufin fluorescence was seen to drop slightly as the amount of ASW increased from 50 to 100%.

Figure 1f, however, shows a potential caveat of interpreting the resazurin data, as the 1-h B. bacteriovorus HD100 population was not negatively affected by the higher osmolalities, as might be assumed based on the resorufin fluorescence values in Fig. 1e. Moreover, there appeared to be a slight but significant increase in the B. bacteriovorus HD100 viabilities in 100% ASW. As no prey were provided in these experiments and this test was performed for only 1 h, however, this could not be due to predation. Taken together, the lack of killing suggests higher osmolalities hinder the metabolism of B. bacteriovorus HD100 and prevent regeneration of the NADH pools without leading to cell death, a result that merits further consideration in a future study. Nevertheless, if true, predation would be inhibited as well, an idea that was explored using 24-h predation tests. As expected, predation by B. bacteriovorus HD100 was inhibited when the ASW content was 25% or higher (Fig. 1g). One unexpected finding was the predator viability trends when the culture was filtered or unfiltered after 24 h. As shown in Fig. 1g, the unfiltered predatory populations were typically 2-log higher in the unpredated cultures, indicating the predator attached to its prey even when the osmolality was very high (1,040 mosmol/kg) but the predation cycle was inhibited. A similar result was found with *Halobacteriovorax* strain JA-1, except it occurred when the osmolality was either very low (40 mosmol/kg) or very high (1,040 mosmol/kg).

**FIG 2:**
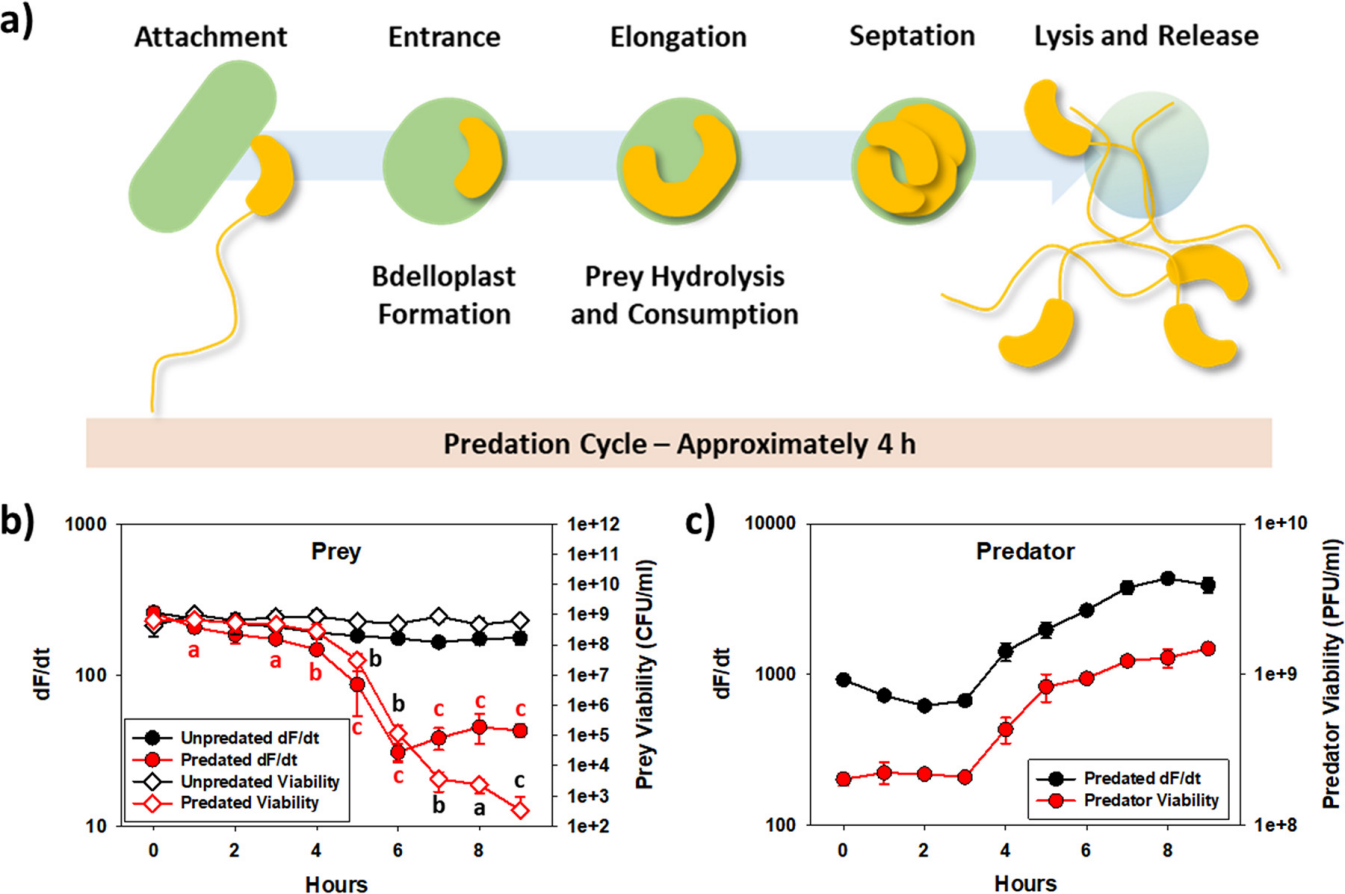
Use of resazurin to monitor predation activities during predation. (a) Predation cycle for B. bacteriovorus HD100. Note one complete cycle typically takes around 4 h. Consequently, predation tests were performed for 9 h to encompass two cycles. (b) Impact of predation on the fluorescence and viability of the prey, E. coli MG1655. The initial predator-to-prey ratio was approximately 0.3, or one predator for three prey. To eliminate the resorufin fluorescence from the predator, SDS was added to the sample aliquots for 10 min prior to adding the resazurin and measuring the dF/dt (*n *= 3). (c) The predator fluorescence (dF/dt) and viability counts paralleled each other. The data show the same trend for both analyses, proving resazurin can be used to quickly assess the predatory population even during predation experiments (*n *= 3).

In a final set of experiments, we sought to apply these principles within a predation test. As the predation cycle typically takes around 4 h (Fig. 2a), this experiment was conducted with an initial predator-to-prey ratio of 0.3, or three prey per predator, and over 9 h to encompass two complete predation cycles. Validating the study by Cho et al. (17), Escherichia coli MG1655 was not sensitive to SDS (see Fig. S2 in the supplemental material), unlike B. bacteriovorus HD100 (Fig. 1d). This difference in sensitivity permitted us to differentiate between the predator and the prey, as shown in Fig. 2b and c. For E. coli MG1655, without predator added, the dF/dt was fairly stable (Fig. 2b), dropping only slightly over time (Fig. S2) but stable on a per-cell basis, as shown in Fig. S3. When the predator was present, however, the dF/dt dropped significantly after 4 h, right when the first predation cycle was expected to finish and the second one to begin (Fig. 2a). As a dead prey would not be able to generate NADH, leading to a lower resorufin fluorescence signal, the rapid loss seen here also validates previous findings, i.e., the prey loses viability rapidly after predation begins (10). When fluorescence at each time point was compared against the corresponding prey viabilities, a strong correlation was observed (Fig. 2b), at least until the dF/dt values leveled out at or near the background (dF/dt = ~30). Similarly, the predatory numbers paralleled the measured dF/dt values when SDS was not added, as shown in Fig. 2c. Moreover, the dF/dt on a per-bacterium basis was stable for B. bacteriovorus HD100 over the extent of the experiment (Fig. S3), showing resazurin can be used to rapidly estimate the predatory concentration even while growing in mixed cultures.

In conclusion, this study demonstrates resazurin as an easy and effective method to rapidly and reliably enumerate predatory viabilities. Its effectiveness was evaluated with evolutionarily different predatory strains and several conditions, including SDS and osmolality, as well as in measuring the predatory populations after a 24-h culture. We also demonstrated resazurin can be coupled with SDS addition to provide information about, and differentiate between, the predator and prey populations in near real-time as the experiment progresses.
