# Supplementary material for: Use of Resazurin To Rapidly Enumerate Bdellovibrio and Like Organisms and Evaluate Their Activities
Source: Microbiol Spectr. 2022 Jun 13;10(3):e00825-22. doi: 10.1128/spectrum.00825-22 (PMC9241754; doi:10.1128/spectrum.00825-22)
Supplement: Supplemental file 1 — Supplemental material. Download spectrum.00825-22-s0001.pdf, PDF file, 0.5 MB [file spectrum.00825-22-s0001.pdf]

Supplemental Information

for

Resazurin to Rapidly Enumerate *Bdellovibrio*-and-Like Organisms (BALOs) and Evaluate Their Activities

Running Title: Quick fluorescence-based estimation of BALO numbers

Hyochan Jang[a], Wonsik Mun[a], Seong Yeol Choi[*] and Robert J. Mitchell[*]

School of Life Sciences, Ulsan National Institute of Science and Technology (UNIST), Ulsan 44919, South Korea

[a] – These authors contributed equally to this study

Contact Information

Email: aster6432@unist.ac.kr (SYC); esgott@unist.ac.kr (RJM)

Tel: +82) 052-217-2513

Table S1. Bacterial strains used in this study

Bacterial Strain	Characteristics
Bdellovibrio bacteriovorus HD100	Bacterial predator – Type-strain
Bdellovibrio bacteriovorus 109J	Bacterial predator
Halobacteriovorax str. JA-1	Bacterial predator isolated from near Jangheung, South Korea
Halobacteriovorax str. JA-2	Bacterial predator isolated from near Jangheung, South Korea
Escherichia coli MG1655/pUCDK	Prey strain for the *Bdellovibrio* predatory strains
Klebsiella pneumoniae NCCP 15782	Clinical pathogen isolated from human blood samples

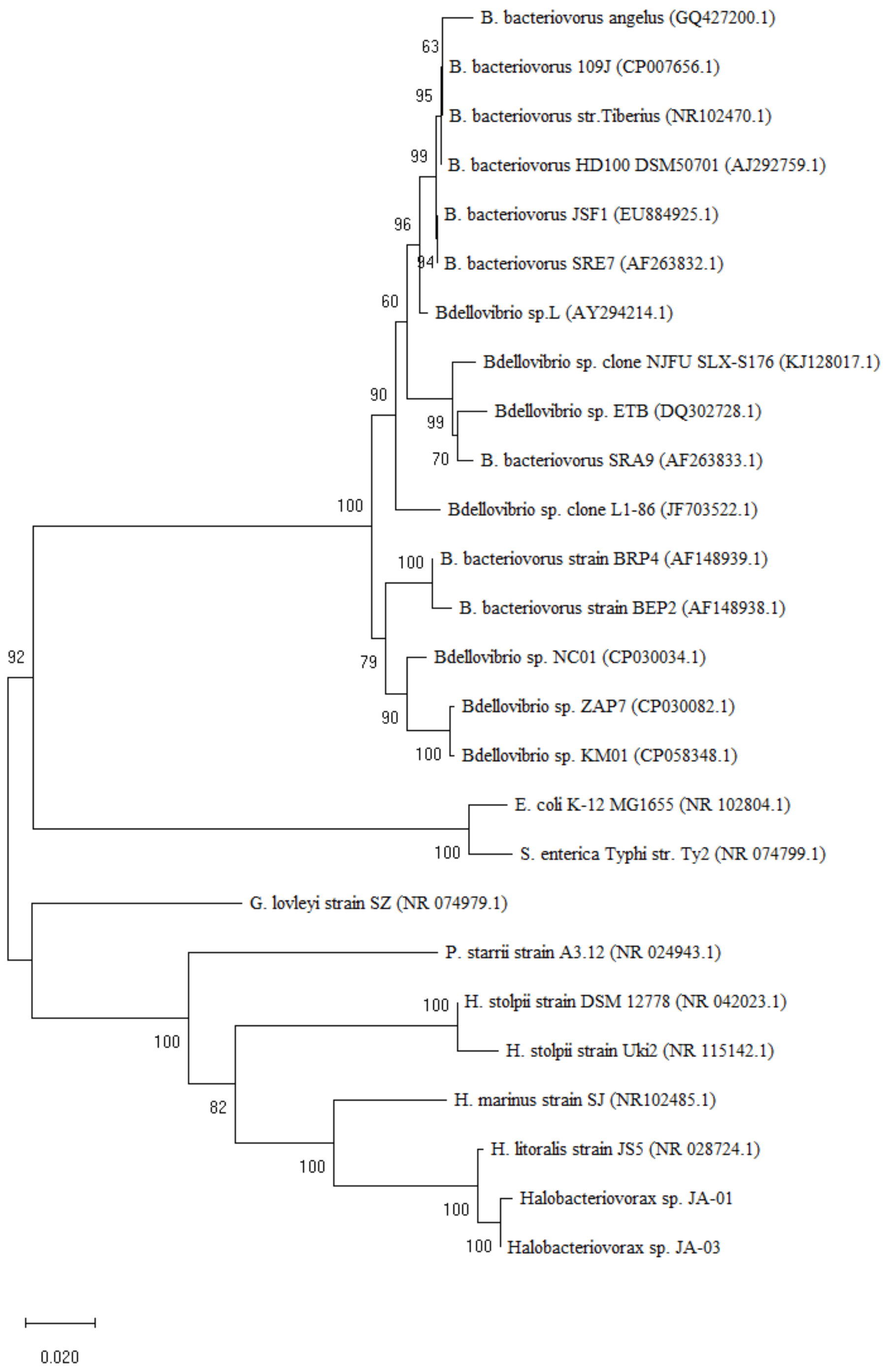

Figure S1. Phylogenetic analyses for *Halobacteriovorax* str. JA-1 and JA-3. The evolutionary history was inferred using the Neighbor-Joining method. The optimal tree with the sum of branch length = 0.67038433 is shown. The percentage of replicate trees in which the associated taxa clustered together in the bootstrap test (2000 replicates) are shown next to the branches.

The tree is drawn to scale, with branch lengths in the same units as those of the evolutionary distances used to infer the phylogenetic tree. The evolutionary distances were computed using the Jukes-Cantor method and are in the units of the number of base substitutions per site. The analysis involved 23 nucleotide sequences. All positions containing gaps and missing data were eliminated. There were a total of 956 positions in the final dataset. Evolutionary analyses were conducted in MEGA7.

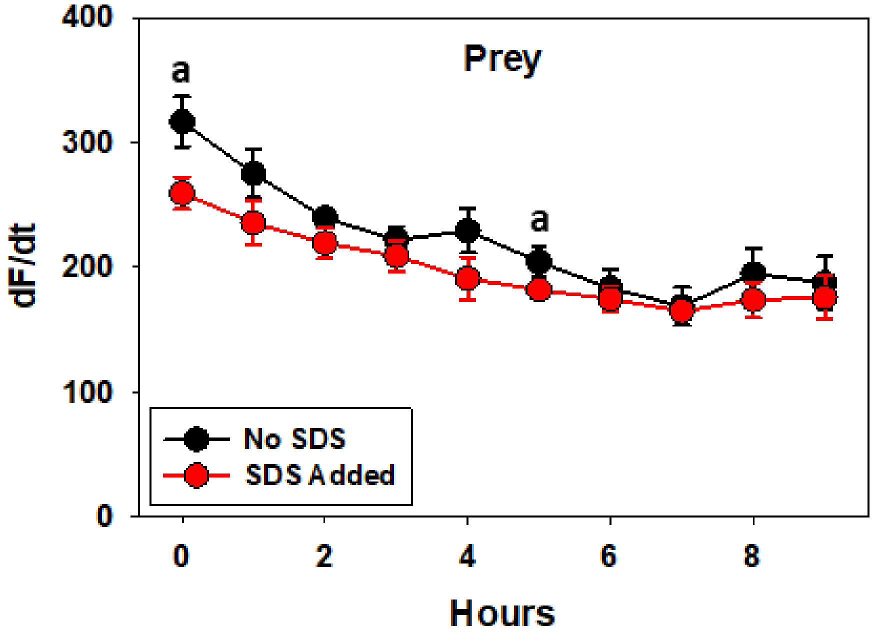

Figure S2. Addition of SDS (0.02% (w:v) final) has only a mild impact of the resorufin fluorescence from *E. coli*, confirming this prey is not negatively impacted by this surfactant.

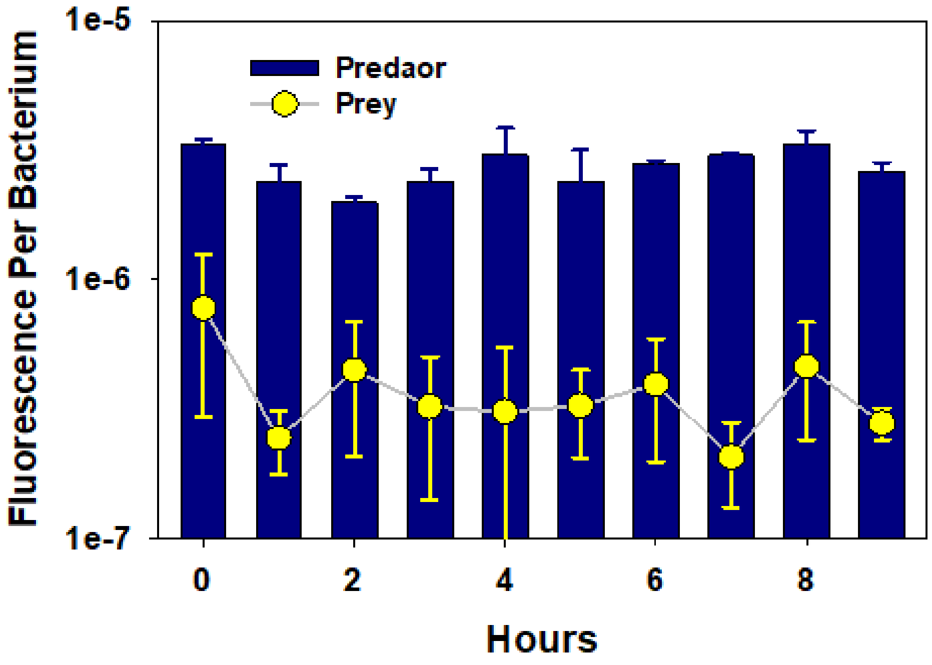

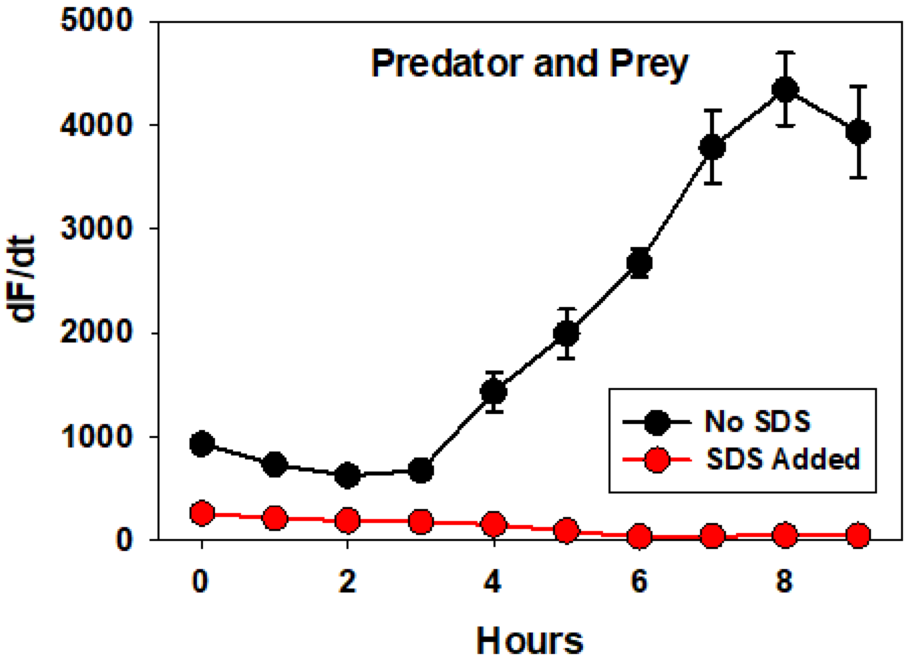

Figure S3. Resorufin fluorescence generated per bacterium, showing the significantly higher (7-fold) fluorescence signal from *B. bacteriovorus* HD100 than *E. coli*. The samples were taken at each hour during the predation tests. For the prey, these samples had no predator or SDS addition. For the predator, the values were calculated based on the difference after SDS addition (Lower). Each was divided by the measured viable counts at each time point. (n = 3)

Materials and Methods

Isolation and Identification of the *Halobacteriovorax* Strains

Samples of the tidal flats were taken south of Jangheung near Sumoon, South Korea. These samples (soil and water) were incubated with shaking at 250 rpm and 30ºC for two hours. After centrifugation (500xg, 5 min) to remove the soil, the supernatant was filtered (0.45 µm syringe filter, Millipore, USA) to remove any large bacterial cells. To the filtrate, *Klebsiella pneumoniae* NCCP 15782, a septicemia-related pathogen, was added to an optical density (600 nm) of 1.0. The cultures were grown under shaking (250 rpm) at 30ºC for several days until the optical density (OD 600 nm) cleared (below 0.2). The cultures were then filtered (0.45 µm syringe filter, Millipore, USA) to remove any remaining prey and bdelloplasts. The filtrate was diluted in 35% artificial seawater (ASW) and added to top agar made with the same media and prey bacterial strain added. The composition of ASW (100%) was described previously (Artificial Seawater (2012)). These plates were incubated at 30ºC for up to a week to allow plaques to form.

Individual plaques were inoculated into sterile 35% artificial seawater (ASW), *i.e.*, with an osmolality between 300 and 350 mOsm/kg, with the same prey to initiate predation once more. Once the culture OD cleared, stocks were prepared and the predator was stored as a bdelloplast formulation in 20% glycerol and -80ºC using the same pathogen as the prey. As needed, fresh plaques of these strains were grown. Subcultures of these isolates were always grown using 30% artificial seawater (ASW; 350 mOsm/kg) with *K. pneumoniae* NCCP 15782 as the prey. After obtaining pure cultures as described above, the newly isolated predators were then identified via 16S rDNA sequencing using the universal primer 27F/1492R set as described previously (Wen et al., 2009; Monnappa et al., 2016) followed by phylogenetic analysis with MEGAversion 6 software (Tamura et al., 2013) (Figure S1).

Growth of the Bacterial Strains

All of the bacterial strains used in this study are listed in Table S1. The prey bacteria used to culture the *B. bacteriovorus* strains was *E. coli* MG1655 as described previously (Monnappa et al., 2014; Im et al., 2018) while *K. pneumoniae* NCCP 15782 was used for the *Halobacteriovorax* isolates. Briefly, each prey was cultivated overnight in Lysogeny Broth (LB) (Difco BD, USA) at 30°C and 250 rpm in a shaking incubator. The prey cells were then pelleted (2,200 x g, 10 min), washed with HEPES buffer (25 mM, pH 7.4) with $CaCl_2$ and $MgCl_2$ added (3 and 2 mM, respectively) and resuspended to an optical density (OD) at 600 nm of 1.0. To these cultures, the predatory bacteria were added (1:100 dilution) and grown in flask cultures. After 24 hours, when the optical density was below 0.2, the predated culture was filtered using 0.45 µM syringe filters (Millipore, USA) to remove any surviving prey and bdelloplasts from the attack-phase predatory cells.

Resazurin Enumeration of the Predatory Bacterial Strains

The resazurin stock (0.2% (w/v)) was prepared using distilled water and filter-sterilized (0.22 µm syringe filter, Millipore, USA). For the experiments, this stock was diluted 1:3 (v:v) into HEPES buffer (25 mM, pH 7.4) with $CaCl_2$ and $MgCl_2$ added (3 and 2 mM, respectively). After growth overnight as described above, the predatory cultures were filtered (0.45 µm syringe filter, Millipore, USA) and serially diluted in either HEPES buffer (*Bdellovibrio*) or 30% ASW (*Halobacteriovorax*). From these dilutions, 200 µl aliquots were transferred into the wells of a black 96-well plate (Greiner, USA) and 20 µl of the diluted resazurin stock were added, giving a final concentration of 0.005% resazurin (w/v). The fluorescence from each well was then immediately read using a plate reader (TECAN, USA; Ex 530nm, Em 580nm). The plate reader was set to mix the samples, maintain the temperature at 30ºC and read the

fluorescence signal in each well every ten minutes. Using the fluorescence values obtained, the change in fluorescence per minute (dF/dt) was calculated manually using the zero- and ten-minute data as follows:

$$\frac{FL_{t=10\ min} - FL_{t=0\ min}}{dt\ (10\ minutes)}$$

SDS Tests

To perform the SDS sensitivity experiments with *B. bacteriovorus* HD100, these experiments were performed as described above and previously (Cho et al., 2019) except the predatory cultures, after 0.45 µm filtration, were diluted ten-fold, *i.e.*, into 180 µl of sterile media (HEPES buffer alone or HEPES buffer with SDS added) 20 µl of the filtered predatory culture were added and mixed within the wells of a black 96-well plate (SPL, Korea). For *Halobacteriovorax* str. JA-1. the same protocol was used except the media used was 25 mM HEPES in 35% ASW. The plates were then incubated at 30 ºC with shaking in a TECAN Spark plate reader. After one hour, 20 µl of the diluted resazurin were added to each well and the fluorescence was measured as described above.

Osmolality tests

The osmolality of the various samples was measured as described previously (Im et al., 2017) using an Advanced 3220 Freezing-Point Osmometer (Advanced Instruments, USA). Briefly, to adjust the osmolality of the samples, artificial seawater (ASW) solutions were mixed with the HEPES preparations to give 0, 25, 50, 75 and 100% ASW.

The short-term effects of the osmolality on the predators were conducted as described above for the resazurin tests. After filtration, the predator was diluted into the different media (1:10) and 200 µl of the culture were incubated in a black 96-well plate (SPL, Korea) at 30 ºC with shaking in a TECAN Spark plate reader. After one hour, 20 µl of the diluted resazurin were added to each well and the fluorescence was measured as described above.

To test the impact of the osmolality on 24-h predation, the prey bacteria were grown as described above, pelleted by centrifugation (13,000 x g, 5 min) and resuspended in the different ASW media at an OD of 1.0. To this, the predatory filtrate was then added (1:100 dilution). These samples were then incubated for 24 hours at 30 ºC with agitation (250 rpm), after which the prey and predator viabilities in each media were determined.

Predation tests

Predation tests were performed using *B. bacteriovorus* HD100 with *E. coli* MG1655 as the prey in 25 mM HEPES buffer (pH 7.2) with 3 mM $MgCl_2$ and 2 mM of $CaCl_2$ added. The prey OD was set to 1.0 and the filtered predator was added so that the initial predator-to-prey ratio was approximately 0.3 (*i.e.*, one predator for three prey). The cultures were incubated at 30 ºC with shaking (250 rpm). Every hour, a sample was taken and used to measure the viabilities using agar plates for the prey and top agar plates for the predator, as described previously (Cho et al., 2019). Briefly, for *B. bacteriovorus* HD100, *E. coli* MG1655 was used as the prey and the top agar consisted of 0.7 % agar prepared using dilute nutrient broth (DNB), *i.e.*, 1/10 NB, with 3 mM $MgCl_2$ and 2 mM $CaCl_2$ added. Similarly, for *Halobacteriovorax* str. JA-1, *K. pneumoniae* NCCP 15782 was used as the prey and the 0.7 % top agar was prepared using DNB with 35 % ASW added. Further additions of $MgCl_2$ and $CaCl_2$ were not needed as they were already present in the ASW.

These hourly aliquots were also tested using resazurin, which we performed without treatment or after a ten-minute treatment with 0.02% SDS. Briefly, 180 µl of the culture were mixed with either 20 µl of HEPES buffer or 20 µl of HEPES with SDS added. After a ten-minute incubation, 20 µl of the diluted resazurin was added and the fluorescence was measured as described above in black 96-well plates (SPL, Korea).

Reproducibility and statistical analysis

Unless specified, each experiment was performed in triplicate and the standard deviations are presented on the graphs as error bars. Normal distribution of each dataset was verified using the Shapiro-Wilk test. For those samples that did not show a substantial departure from the normality ($p > 0.05$), the student t-test was used, with significance indicated within the graphs using: *a* - $p < 0.05$; *b* - $p < 0.01$; *c* - $p < 0.001$. For the resorufin fluorescence per predatory density, Shapiro-Wilk found them to be significant ($p < 0.05$) and, thus, the correlations (R^2 values) between the predatory density and the resorufin fluorescence were calculated using the Spearman Correlation Test.

References

Artificial Seawater. Cold Spring Harbor Protocols 2012;2012(2).

Cho G, Kwon J, Soh SM, Jang H, Mitchell RJ. Sensitivity of predatory bacteria to different surfactants and their application to check bacterial predation. Applied Microbiology and Biotechnology 2019;103(19):8169-8178.

Im H, Dwidar M, Mitchell RJ. Bdellovibrio bacteriovorus HD100, a predator of Gram-negative bacteria, benefits energetically from Staphylococcus aureus biofilms without predation. ISME J 2018;12(8):2090-2095.

Im H, Son S, Mitchell RJ, Ghim C-M. Serum albumin and osmolality inhibit Bdellovibrio bacteriovorus predation in human serum. Scientific Reports 2017;7(1).

Monnappa AK, Bari W, Choi SY, Mitchell RJ. Investigating the Responses of Human Epithelial Cells to Predatory Bacteria. Scientific Reports 2016;6(1).

Monnappa AK, Dwidar M, Seo JK, Hur JH, Mitchell RJ. Bdellovibrio bacteriovorus inhibits Staphylococcus aureus biofilm formation and invasion into human epithelial cells. Sci Rep 2014;4:3811.

Tamura K, Stecher G, Peterson D, Filipski A, Kumar S. MEGA6: Molecular Evolutionary Genetics Analysis Version 6.0. Molecular Biology and Evolution 2013;30(12):2725-2729.

Wen CQ, Lai XT, Xue M, Huang YL, Li HX, Zhou SN. Molecular typing and identification ofBdellovibrio-and-like organisms isolated from seawater shrimp ponds and adjacent coastal waters. Journal of Applied Microbiology 2009;106(4):1154-1162.
